# In Spare Moments

**Published:** 1889-01-19

**Authors:** 


### In Spare Moments.

Some time ago, a lady who is at the head of a type-writing establishment received some copying to do. The subject of the MS. entrusted to her was the necessity for hospitals, and the good work done by them. Now, it is well known that the average copyist is almost as mechanical as the type-writer itself; and one would as soon expect to hear the "Remington" lift up its voice and speak as to learn that a copyist had taken the least interest in the matter he was transcribing. We have spoken of the average copyist as masculine; perhaps it was because Madame Monchablon, the lady in question, is of the inferior sex, and employs a staff of women, that neither she nor they have been able to attain this high ideal of indifference. Whatever be the explanation, they took an interest in their work. They learned a good deal about hospitals in copying those pages. They learned that nearly one-fourth of the population of London needs medical help gratuitously, or at least at rates that cannot be remunerative to those who give it. They learned that, in spite of all our hospitals, there are hundreds who cannot be attended to because there are no beds for them. They learned that—sadder still—even when there are vacant beds, there is not always money to permit of their being occupied.

From this arose a personal interest in hospitals and their occupants, and a desire on the part of all these girls to do what they could for them. Perhaps some of them had been in hospital themselves and knew what it was—knew that in spite of unwearying care and attention, the hours sometimes seem long, and little tokens from the outer world are valued; therefore they wanted to send some little gifts of remembrance to their unknown brothers and sisters; especially to the little brothers and sisters—the children on whom the necessary confinement hangs heaviest. Therefore at Christmas time they occupied their spare moments in dressing a number of "Kate Greenaway" dolls, and preparing books of nursery rhymes, which they sent to the editor of The Hospital to give away where he thought best.

These books are altogether unique. The leaves are of linen, worked round to prevent fraying out, and the poems are printed with the type-writer, so that no duplicate copies are to be had for love or money. It is well-known that books of which the edition is small are very valuable, and large prices are given for them by bibliophiles. What should be the value of these books in which the edition is strictly limited to one? What the market value would be we do not know, but this is certain, that to the languid and suffering children who will receive them their value will be priceless.

And of this also are we certain, that if we were a hospital official and wanted any type-writing done, we would send it all to Madame L. Monchablon, 23, Austinfriars, E.C., under whose kindly supervision these unique books were produced. We have had great pleasure in sending them, together with the dolls, to the matron of the Home for Incurable Children, Maida Vale, W.

